# Morphometric dataset of *Varanus salvator* for non-invasive sex identification using machine learning

**DOI:** 10.1038/s41597-024-03172-9

**Published:** 2024-04-05

**Authors:** Ariff Azlan Alymann, Imann Azlan Alymann, Song-Quan Ong, Mohd Uzair Rusli, Abu Hassan Ahmad, Hasber Salim

**Affiliations:** 1 Barn Owl and Rodent Research Group, School of Biological Sciences, Universiti Sains Malaysia, 11800 Pulau Pinang, Malaysia; 2 Institute for Tropical Biology and Conservation, Universiti Malaysia Sabah, Jalan UMS, 88400 Kota Kinabalu, Sabah Malaysia; 3 Sea Turtle Research Unit (SEATRU), Institute of Oceanography and Environment (INOS), Universiti Malaysia Terengganu, 21030 Kuala Nerus, Terengganu Malaysia

**Keywords:** Herpetology, Animal physiology

## Abstract

Reliable sex identification in *Varanus salvator* traditionally relied on invasive methods like genetic analysis or dissection, as less invasive techniques such as hemipenes inversion are unreliable. Given the ecological importance of this species and skewed sex ratios in disturbed habitats, a dataset that allows ecologists or zoologists to study the sex determination of the lizard is crucial. We present a new dataset containing morphometric measurements of *V. salvator* individuals from the skin trade, with sex confirmed by dissection post- measurement. The dataset consists of a mixture of primary and secondary data such as weight, skull size, tail length, condition etc. and can be used in modelling studies for ecological and conservation research to monitor the sex ratio of this species. Validity was demonstrated by training and testing six machine learning models. This dataset has the potential to streamline sex determination, offering a non-invasive alternative to complement existing methods in *V. salvator* research, mitigating the need for invasive procedures.

## Background & Summary

The study of morphological differences between the sexes in *Varanus salvator* is ecologically important, especially considering that the species is extensively used for the skin trade and that anthropogenic habitat disturbance is thought to influence the sex ratio^1,2^. Surprisingly, little attention has been paid to sex determination in *V. salvator* based on morphometric proportions of the body, although the general body morphology of the species has been extensively studied^3–5^. Within varanids, species show considerable variation in body size, with larger species often exhibiting more pronounced sexual dimorphism^6^, which is consistent with Rensch’s rule^7^. Specific features such as variations at the base of the tail (where the male hemipenes are located) and the proportions of the head shape have also been reported^8,9^.

Reliable sex determination of *V. salvator* in the field would facilitate the measurement of sex ratios, which is crucial for drawing conclusions about population dynamics in disturbed habitats. Currently, unambiguous sexing requires invasive methods in which the reproductive organs are measured during dissection or genetic analysis^9,10^. Less invasive methods, such as hemipenis inversion, are unreliable due to the possible for confusion between partially elongated male hemipenis and female hemiclitori but are still used in ecological studies^1^. Previous studies suggest that tail-to-body ratio, eye-to-ear length, and the extent of the tail base are potential features for sex determination in this species^4,9,11^. Therefore, there are many research questions that need to be answered by investigating the relationship between the sex of *V. salvator* and its morphology, and a dataset that allows statistical or machine learning modelling is crucial.

We present a morphometric dataset that provides a non-invasive method for sex prediction and can potentially improve the accuracy of sex determination in the field alongside the commonly practised hemipenes inversion. This dataset is useful for various fields, including machine learning engineers, app developers, data scientists, ecologists, herpetologists, conservationists, and others.

## Methods

### Sampling

This study sampled a total of 146 individual *V. salvator*; 83 females, 63 males. Lizards were sampled in a skin factory in Johor (location provided by Department of Wildlife and National Parks Peninsular Malaysia (PERHILITAN)). All lizards were sourced from oil palm plantations in Perak. The sample size was determined by the allocation provided to the researchers by the skin factory.

### Dataset formation

Lizard morphological features measured included the following: thigh width (TW), base tail circumference (BTC), skull length (SL), skull width (SW), eye to ear length (EEL), snout-vent length (SVL), snout-tail length (STL), tail length (TL), and weight^1,3,5,9,11,12^. Length measurements were made using a flexible measuring tape whereas weight measurements were made using a handheld weighing scale. From the measurements made above, TW, BTC, SL, SW, EEL, and TL were divided by STL to derive relative proportions. Similarly, SW and EEL were divided by SL to derive relative head proportions. These variables were used for analysis, as some of the literature suggests relative proportions in body morphology and head dimension could be different between sexes^3,4,8^. Body condition was made by dividing weight by STL, similar to a body mass index^1^. Body size assessment involved a principal components analysis (PCA) performed on eight morphometric variables, namely TW, BTC, SL, SW, EEL, SVL, STL, and weight (similar to^13^). Component number 1 from the resulting PCA output was subsequently utilized as body size (Tables 1 and 2). Definitions of morphometric variables used for sex prediction are provided in Table 3.

**Table 1:** KMO and Bartlett’s test results indicating variables are suitable for PCA.

KMO	0.951	
Bartlett’s test of sphericity	χ ^2^_(15)_ = 1225.159	p < 0.001

**Table 2:** Eigenvalue and percentage of variance explained for all components.

Component Number	Eigenvalue	Percentage of Variance Explained
1	6.288	78.599
2	0.428	5.346
3	0.322	4.020
4	0.270	3.369
5	0.245	3.059
6	0.201	2.518
7	0.136	1.699
8	0.111	1.389
	Total Percentage	100

**Table 3:** Definition of morphometric variables used for sex prediction.

No.	Variable	Definition	Type
1.	Body condition	Weight divided by STL (kg^cm^)	Continuous
2.	Base tail circumference (BTC)	Circumference of the tail after the cloaca (cm)	Continuous
3.	BTC: STL	BTC divided by STL	Continuous
4.	Body size	PCA output of 8 morphometric variables	Continuous
5.	Eye to ear length (EEL)	Length from the anterior tip of the eye to the posterior tip of the ear (cm)	Continuous
6.	EEL: Skull Length	EEL divided by Skull Length	Continuous
7.	EEL: STL	EEL divided by STL	Continuous
8.	Skull length (SL)	Length from the tip of the snout to the base of the skull (cm)	Continuous
9.	SL: STL	SL divided by STL	Continuous
10.	Skull width (SW)	Length of the broadest part of skull (cm)	Continuous
11.	SW: STL	SW divided by STL	Continuous
12.	SW: SL	SW divided by SL	Continuous
13.	Snout-tail length (STL)	Length form tip of snout to end of tail (cm)	Continuous
14.	Snout-vent length (SVL)	Length from tip of snout to vent (cloaca) (cm)	Continuous
15.	Tail length (TL)	Length of tail from cloaca to tip (cm)	Continuous
16.	TL: STL	TL divided by STL	Continuous
17.	Thigh width (TW)	Circumference of thigh at middle of thigh (cm)	Continuous
18.	TW: STL	TW divided by STL	Continuous
19.	Weight	Weight of animal (kg)	Continuous
20.	Sex	Male = 0, Female = 1	Categorical

### Ethics statements

All authors confirm that we have complied with all relevant ethical regulations. A permit to conduct research on this species has been secured from PERHILITAN, license number P-00003-15-19; as well as animal ethics approval from Universiti Sains Malaysia, Animal ethics approval number USM/IACUC/2020/(123)(1064).

## Data Records

The dataset is publicly available on Figshare at the link: 10.6084/m9.figshare.24558595^14^. Morphometric measurements were categorised according to sex (83 females, 63 males). The raw data were recorded in a physical data sheet predefined with the attributes and digitised into an Excel file and saved in CSV format. The data were checked, cleaned, and processed into independent variables that can serve as predictors and dependent variables according to the lizards’ sex.

## Technical Validation

### Pilot testing with basic model construction

A pilot study was conducted to validate the suitability of the dataset in predicting the sex of *V. salvator*. Six machine learning models were used: logistic regression, random forest, support vector machine, extreme gradient boosting, adaptive boosting, and gaussian naïve bayes. For training and validation, data were split 70% for training, and 30% for validation. Model construction, training and validation was conducted using Python programming in Google Colab workbook. The resulting confusion matrixes and model performances are summarized in Supplementary Table 1.

## Usage Notes

This dataset contains morphological measurements form 83 females, 63 male *V. salvator*. However, it is important to acknowledge several limitations inherent to the dataset. Firstly, the data predominantly represents smaller individuals, as it was collected from individuals captured for the skin trade. Skin factories typically accept individuals weighing ≤5 kg, contributing to this size bias. Additionally, individuals from other habitats like forests and urban areas are notably absent from this dataset, given that the data collection exclusively pertained to animals sourced from oil palm plantations. Moving forward, to enhance the applicability of morphological data analysis, it is recommended to include individuals from wild populations in model training and validation. This inclusion could lead to the development of an app where inputting relevant morphological variables can determine the sex of wild individuals, allowing for easy sex identification in the field. Furthermore, future work could explore image-based means of sex identification, which could prove more time and cost efficient to conduct.

### Supplementary information

Supplementary Table 1

## Data Availability

The Python code utilised in this study is available on Kaggle, via the link: https://www.kaggle.com/code/ariffazlanalymann/ml-varanus-morphology-sex-prediction.
