# Supplementary material for: Morphometric dataset of Varanus salvator for non-invasive sex identification using machine learning
Source: Sci Data. 2024 Apr 5;11:337. doi: 10.1038/s41597-024-03172-9 (PMC10997747; doi:10.1038/s41597-024-03172-9)
Supplement: Supplementary file 1 — Supplementary Table 1 [file 41597_2024_3172_MOESM1_ESM.docx]

Supplementary Table 1

| **Machine learning models** | **Confusion matrix** | **Model parameters** | | | |
| --- | --- | --- | --- | --- | --- |
|  |  | **Accuracy** | **Precision** | **Sensitivity** | **F1 value** |
| **Logistic Regression** | 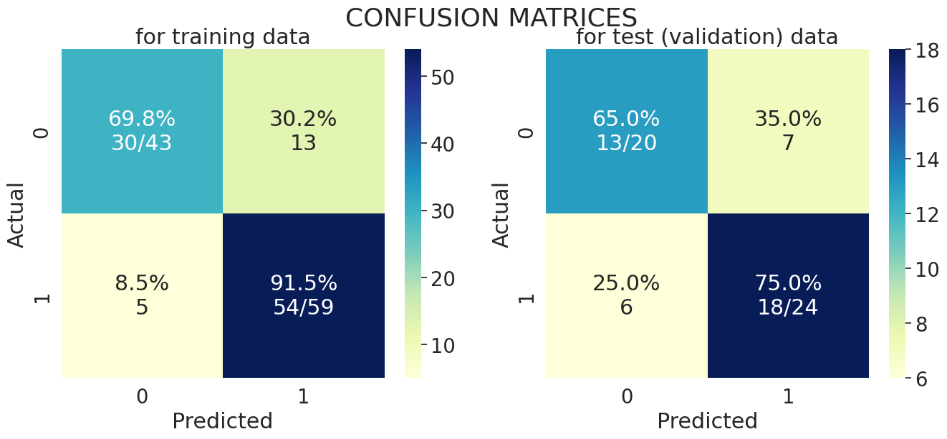  (C: 0.2) | 0.7045 | 0.7200 | 0.7500 | 0.7347 |
| **Random Forest** | 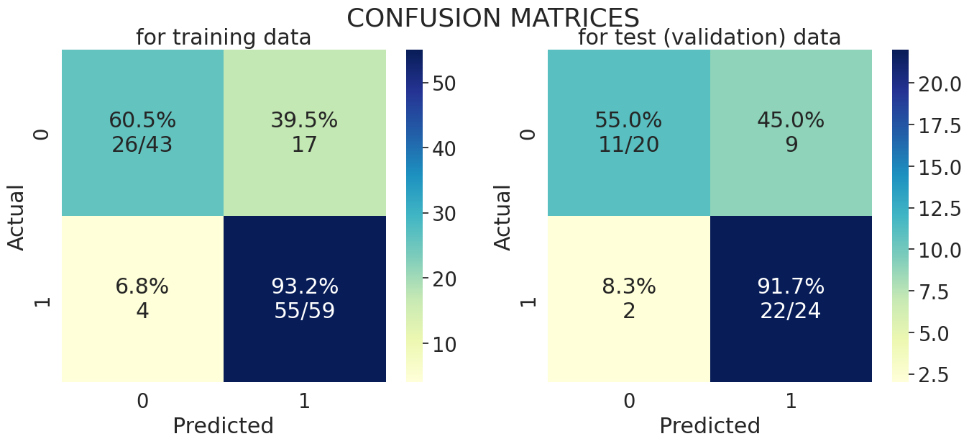  (criterion: gini) | 0.7500 | 0.7097 | 0.9167 | 0.8000 |
| **Support Vector Machine** | 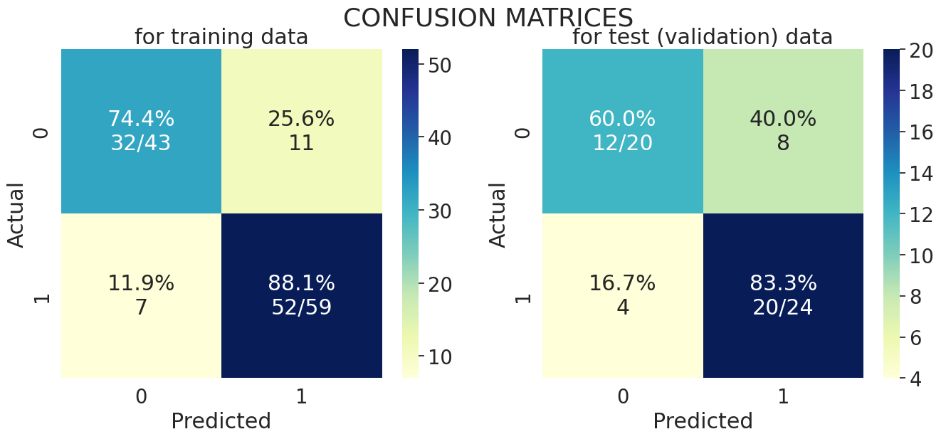  (Kernel: Linear, tol:0.001) | 0.7273 | 0.7143 | 0.8333 | 0.7692 |
| **Extreme Gradient Boosting** | 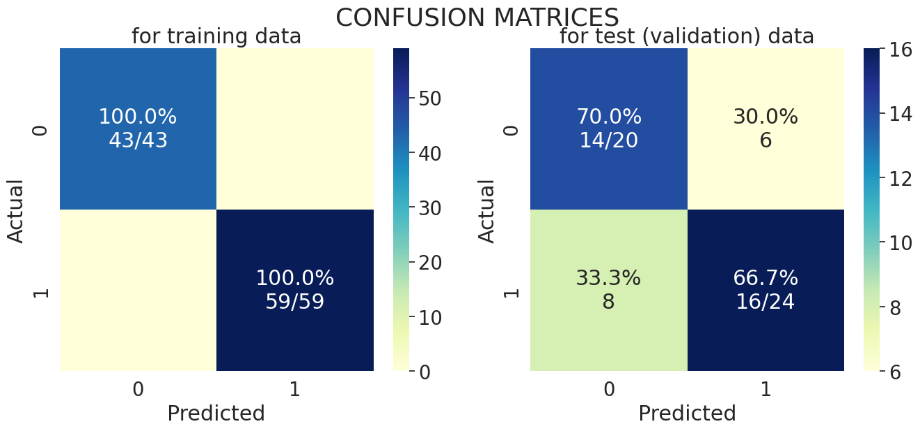  (Learning rate: 0.09) | 0.6818 | 0.7273 | 0.6667 | 0.6957 |
| **Adaptive Boosting** | 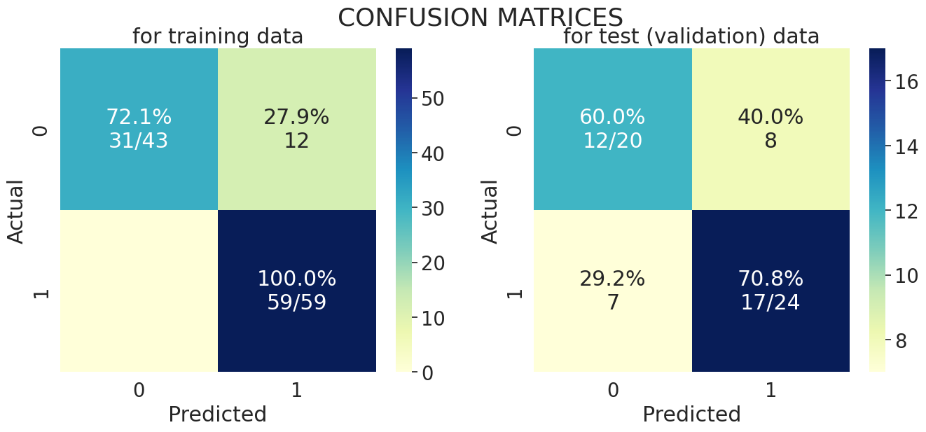  (Learning rate: 0.105) | 0.6591 | 0.6800 | 0.7083 | 0.6939 |
| **Gaussian Naïve Bayes** | 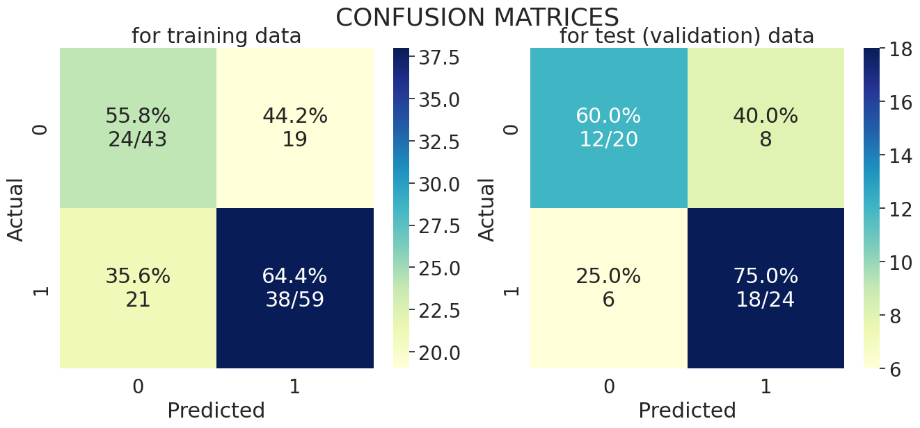  (Var smoothing: 0.0001) | 0.6818 | 0.6923 | 0.7500 | 0.7200 |
| legend | 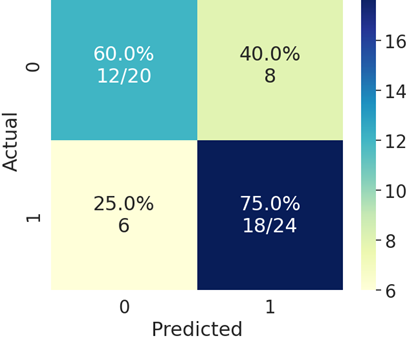 |  |  |  |  |
